# Parameters That Enhance the Bacterial Expression of Active Plant Polyphenol Oxidases

**DOI:** 10.1371/journal.pone.0077291

**Published:** 2013-10-21

**Authors:** Mareike E. Dirks-Hofmeister, Stephan Kolkenbrock, Bruno M. Moerschbacher

**Affiliations:** Department of Plant Biology and Biotechnology, Westphalian Wilhelms-University of Münster, Münster, Germany; Centro Nacional de Biotecnologia – CSIC, Spain

## Abstract

Polyphenol oxidases (PPOs, EC 1.10.3.1) are type-3 copper proteins that enzymatically convert diphenolic compounds into their corresponding quinones. Although there is significant interest in these enzymes because of their role in food deterioration, the lack of a suitable expression system for the production of soluble and active plant PPOs has prevented detailed investigations of their structure and activity. Recently we developed a bacterial expression system that was sufficient for the production of PPO isoenzymes from dandelion (*Taraxacum officinale*). The system comprised the *Escherichia coli* Rosetta 2 (DE3) [pLysSRARE2] strain combined with the pET-22b(+)-vector cultivated in auto-induction medium at a constant low temperature (26°C). Here we describe important parameters that enhance the production of active PPOs using dandelion PPO-2 for proof of concept. Low-temperature cultivation was essential for optimal yields, and the provision of CuCl_2_ in the growth medium was necessary to produce an active enzyme. By increasing the copper concentration in the production medium to 0.2 mM, the yield in terms of PPO activity per mol purified protein was improved 2.7-fold achieving a v_max_ of 0.48±0.1 µkat per mg purified PPO-2 for 4-methylcatechol used as a substrate. This is likely to reflect the replacement of an inactive apo-form of the enzyme with a correctly-folded, copper-containing counterpart. We demonstrated the transferability of the method by successfully expressing a PPO from tomato (*Solanum lycopersicum*) showing that our optimized system is suitable for the analysis of further plant PPOs. Our new system therefore provides greater opportunities for the future of research into this economically-important class of enzymes.

## Introduction

**Figure 1:**
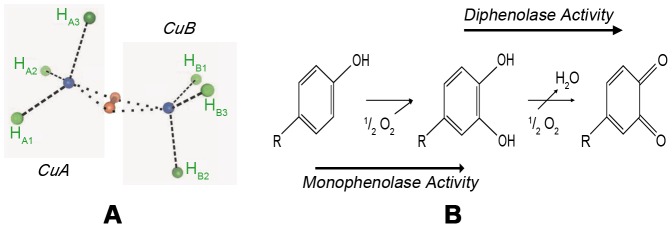
Structure and catalysis of PPOs and related enzymes. A Type-3 copper center. Conserved histidines of the two copper centers (CuA and CuB) are labeled (H_A1_-H_A3_, H_B1_-H_B3_) and colored in green. Copper is colored blue and the oxygen molecules of the bound peroxide in red (*modified from* [31]). **Phenoloxidase activities.** Tyrosinases oxidase monophenolic compounds via their monophenolase activity to diphenols and further to the respective quinones. PPOs mostly just catalyze the conversion of diphenols to quinones (diphenolase activity).

Polyphenol oxidases (PPOs, EC 1.10.3.1) are type-3 copper enzymes (Figure 1A) found in most plants [1], [2]. They are closely related to tyrosinases (EC 1.14.18.1) and catalyze the oxygen-dependent conversion of *o*-diphenols to *o*-quinones (diphenolase activity, Figure 1B). PPOs are of great interest in the food industry because they accelerate the browning of fruits and vegetables during handling and storage [3]–[5].

Despite intensive research focusing on the characterization of plant PPOs, functional analysis has been hampered by inefficient enzyme purification. This is caused by (a) the tight regulation of production, which limits the abundance of PPOs in vivo, (b) PPO aggregation with phenolic compounds during extraction, and activation by proteolysis, both of which limit the quality and quantity of the recovered enzyme, and (c) the multiplicity of isoforms, including different isoenzymes and differently-processed forms of the same enzyme, which often leads to cross-contamination. The successful heterologous expression of plant PPOs in *Escherichia coli* to avoid these issues has been reported only rarely [6]–[8], indicating problems associated with bacterial production [1], [9]. Typically, the recombinant enzyme is either soluble but shows only residual activity [6], or it is mainly found in the insoluble fraction [7]. There is little information available about attempts to optimize PPO production in bacteria, making it difficult to develop a reliable and effective expression strategy.

The inefficient expression of plant PPOs in bacteria may in part be caused by inappropriate codon usage but probably also by the presence of two strictly-conserved intramolecular disulfide bonds and an uncommon thioether bridge between specific cysteine and histidine residues [10]–[12]. The enzyme is unlikely to fold properly in the absence of these bonds [13] but the *E. coli* cytoplasm provides an unfavorable environment for this process. Furthermore, plant PPOs must incorporate copper, but a suitable copper chaperone may not be present in *E. coli*. Toxicity of PPOs towards bacteria also cannot be ruled out [14], [15]. In contrast to these potential drawbacks, PPOs also possess certain favorable properties for expression in bacteria, including the absence of introns in many plant PPO genes [1], [2] and the translocation of the protein into the thylakoid lumen via the twin-arginine translocation pathway [16], which suggests that the protein folds (without the need for post-translational modifications such as glycosylation) in the chloroplast stroma, an environment similar to that of the bacterial cytoplasm.

**Figure 2:**
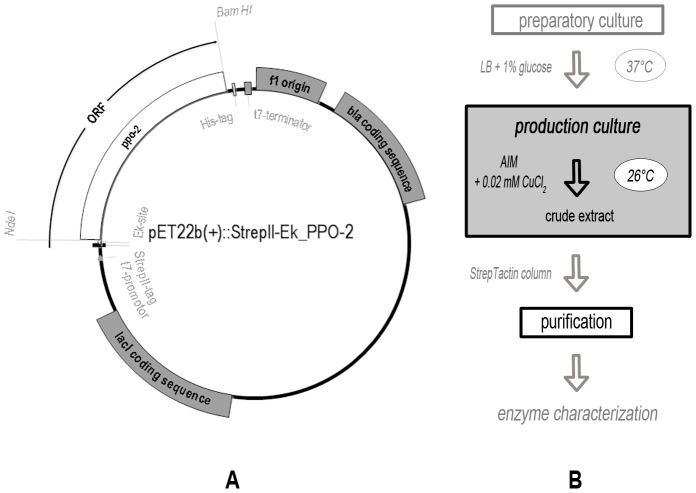
Expression system for dandelion PPOs. A Scheme for the PPO-2 expression plasmid. Location of important genes, regulatory elements and sites for restriction enzymes are given. The StrepII-tag (WSHPQFEK) and the Enterokinase recognition site (Ek-site: DDDDK) were introduced via PCR. *ORF: open reading frame*. **B Procedure of heterologous PPO-expression.** Conditions like media, supplements and temperatures are given and the course of action is schematized. *AIM: autoinduction medium; LB: lysogeny broth*.

We recently introduced a method for the efficient production of large amounts of pure, active and soluble PPOs from dandelion (*Taraxacum officinale*) in *E. coli* Rosetta 2 (DE3) [pLysSRARE2] cells [17]. We included several modifications to promote folding, such as an N-terminal StrepII-tag that allows detection and purification without interfering with folding and also avoids product loss by C-terminal proteolysis, which is typical for PPOs [18]. Production cultures (Figure 2B) were grown in autoinduction medium (AIM), which avoids the need for manual induction and allows the production of toxic proteins when sufficient biomass has been produced [19]. The AIM was supplemented with small amounts of divalent copper as previously suggested to encourage correct folding of the type-3 copper center [6], and the cells were cultivated at low temperatures (26°C) also to encourage proper folding [20]. This protocol has been sufficient for the production of all dandelion PPO isoenzymes that have been tested thus far.

To further improve the quantity and quality of recombinant PPO protein obtained, we set out to optimize some of the cultivation parameters necessary for the successful production of PPOs, by maximizing the yields and activity of recombinant dandelion PPO-2 (ToPPO-2). We also tested the transferability of the optimized system to additional plant PPOs to promote future research on the structure-function relationships among type-3 copper proteins.

## Materials and Methods

### Cloning

We cloned the PPO sequences listed in Table 1 by PCR using gene-specific primers that also introduced appropriate restriction sites, and recovered the native sequences encoding the mature PPOs (lacking the transit peptides). The genes were ligated with the vector pET-22b(+) (Novagen®, Merck, Germany), bearing an upstream located StrepII-tag encoding sequence as shown in Figure 2 and *E. coli* TOP10 cells were transformed with these vectors. All constructs were verified by sequencing before introduction into *E. coli* Rosetta 2 (DE3) [pLysSRARE2] (Novagen®, Merck, Germany).

**Table 1:** PPO genes and associated cloning strategies.

notation	source	primer sequences (*introduced restriction sites are underlined*)	accession no.	cloning background			product information		
				size	5′site	3′site	ORF	M_W (native)_	M_W (tagged)_
**ToPPO-2**	*Taraxacum officinale*	fw 5′CAT ATG GAC CCG ATC ATG GCA CCC G′3; rev 5′-GGA TCC TCA ATC CTC ATA CTC GAT TTT CAT CC-3′	**FM178478**	1515 bp	NdeI	BamHI	505 aa	56.8 kDa	**58.9 kDa**
**ToPPO-6**	*Taraxacum officinale*	fw 5′CAT ATG GAC CCA ATC ACC ACC CCC GAC ATT TCC-3′; rev 5′AAG CTT CAC TCC TCA ATA GGA ACT AAC TCG-3′	**FR863688**	1539 bp	NdeI	HindIII	513 aa	57.8 kDa	**60.0 kDa**
**SlPPO-F**	*Solanum lycopersicum*	fw 5′-CAT ATG GCT CCT ATA CCA CCA CC-3′; rev 5′-GTC GAC TTT AAC AAT CCT CAA GCT TG-3′	**Z12838**	1503 bp	NdeI	SalI	500 aa	57.0 kDa	**59.2 kDa**

### Heterologous Expression

Preparatory cultures were cultivated in 50-ml Falcon tubes containing 20 ml of LB medium supplemented with appropriate antibiotics and 1% glucose to prevent induction. These cultures were inoculated from cultures grown on solid medium and then cultivated at 37°C for ∼4 h until the OD_600 nm_ reached a value between 0.5 and 1, indicating the start of the exponential growth phase.

Production cultures were inoculated to an OD_600 nm_ of 0.02 by diluting the preparatory culture appropriately. The AIM comprised LB medium supplemented with solution 5052 (50× stock: 25% glycerol, 2.5% glucose, 10% α-lactose monohydrate), mineral stock M (50× stock: 1.25 M Na_2_HPO_4_, 1.25 M KH_2_PO_4_, 2.5 M NH_4_Cl, 0.25 M Na_2_SO_4_), appropriate antibiotics and 2 mM MgSO_4_ [19]. Under standard conditions, 30 ml cultures were supplemented with 0.02 mM CuCl_2_ and grown at 26°C for 48 h under shaking at 120 rpm. In some experiments, the amount of CuCl_2_ and the cultivation temperature were varied to determine optimal conditions. To maximize reproducibility and facilitate the interpretation of results, we prepared a master mix solution for the production cultures in each independent experiment so that only the test parameter was varied.

### Sampling, Crude Protein Extracts and Determination of Protein Content

Samples were taken from production cultures when necessary to evaluate specific parameters. Bacterial growth was determined by measuring the OD_600nm_ of cultures diluted 1:10 in water. For further analysis, 1 ml of culture was centrifuged (∼12,000×*g*, 10 min, 4°C) and the pellet was resuspended in 100 µl of 100 mM maleate-Tris buffer (pH 6.0) and frozen. Crude extracts were prepared as stated below.

After 48 h, whole cultures were harvested by centrifugation as above. Pellets were resuspended in 0.1 volume of 100 mM maleate-Tris buffer (pH 6.0) and frozen. The cells lysed automatically when they thawed, and the DNA was digested with benzonase (Novagen®, Merck, Germany). A crude protein extract was recovered by centrifugation (∼5000×*g*, 10 min, 4°C) and used for the further characterization and purification of the PPOs. The protein concentration was determined using the Bradford assay [21].

### PPO Purification

PPOs were purified by affinity chromatography using Strep-Tactin columns (1 ml bed volume; Qiagen, Hilden, Germany) in 50 mM Tris buffer (pH 8.0) containing 400 mM NaCl at 4°C. After elution, PPOs were re-buffered in 100 mM maleate-Tris buffer (pH 6.0) and used within 24 h for activity analysis. Samples for SDS-PAGE and copper analysis were stored up to several days at 4°C.

### PPO Activity and Enzyme Kinetics

PPO activity and kinetics were investigated as previously described [17] using 4-methylcatechol as a standard substrate. The maximum slope of product formation was converted to activity by including the specific extinction coefficient (ε_405 nm_ = 1090 M^-1^cm^-1^) of the quinone [17]. PPO activity under standard conditions was measured using a total of 5 µg protein in crude extracts or 1 pmol purified PPO per 200 µl reaction volume in 50 mM acetate-phosphate buffer (pH 6.0) supplemented with 0.75 mM SDS to induce PPO-activity [1], [22]–[24].

### Enterokinase digestion to cleave the StrepII-tag

To test the influence of the StrepII-tag on PPO activity, 0.3 mg/ml purified PPO was incubated at 4°C with 0.1% (w/w) of Enterokinase (New England BioLabs, Ipswich, USA) in 100 mM maleate-Tris buffer (pH 6.0) for 16 h (Figure S1).

No influence of the tag was observed, and all reported experiments where performed using the fusion proteins.

### SDS-PAGE Analysis and Western Blotting

Samples were boiled for 10 min in reducing loading dye (62.5 mM Tris/HCl pH 6.8, 2% SDS, 5% glycerol, 0.04% bromophenol blue, 1 mM DTT), separated by SDS-PAGE on 12% acrylamide gels [25] and stained using 0.1% Coomassie-Brilliant-Blue G-250 in 40% methanol, 5% acetic acid.

Proteins were transferred from the acrylamide gels to a nitrocellulose membrane and recombinant PPOs carrying a StrepII-tag were detected by chemiluminescent western blot using Strep-Tactin labeled with horseradish peroxidase (Strep-Tactin® HRP-conjugate) according to the manufacturer's instructions (IBA, Germany).

### Quantification of copper by TXRF

Total reflection X-ray fluorescence analysis (TXRF) was carried out using a S2-PICOFOX fluorometer (Bruker AXS, Germany) as previously described [26], with settings provided earlier [17].

## Results and Discussion

### Expressing ToPPO-2 at 26°C for 48 h in AIM supplemented with 0.02 mM CuCl_2_

**Figure 3:**
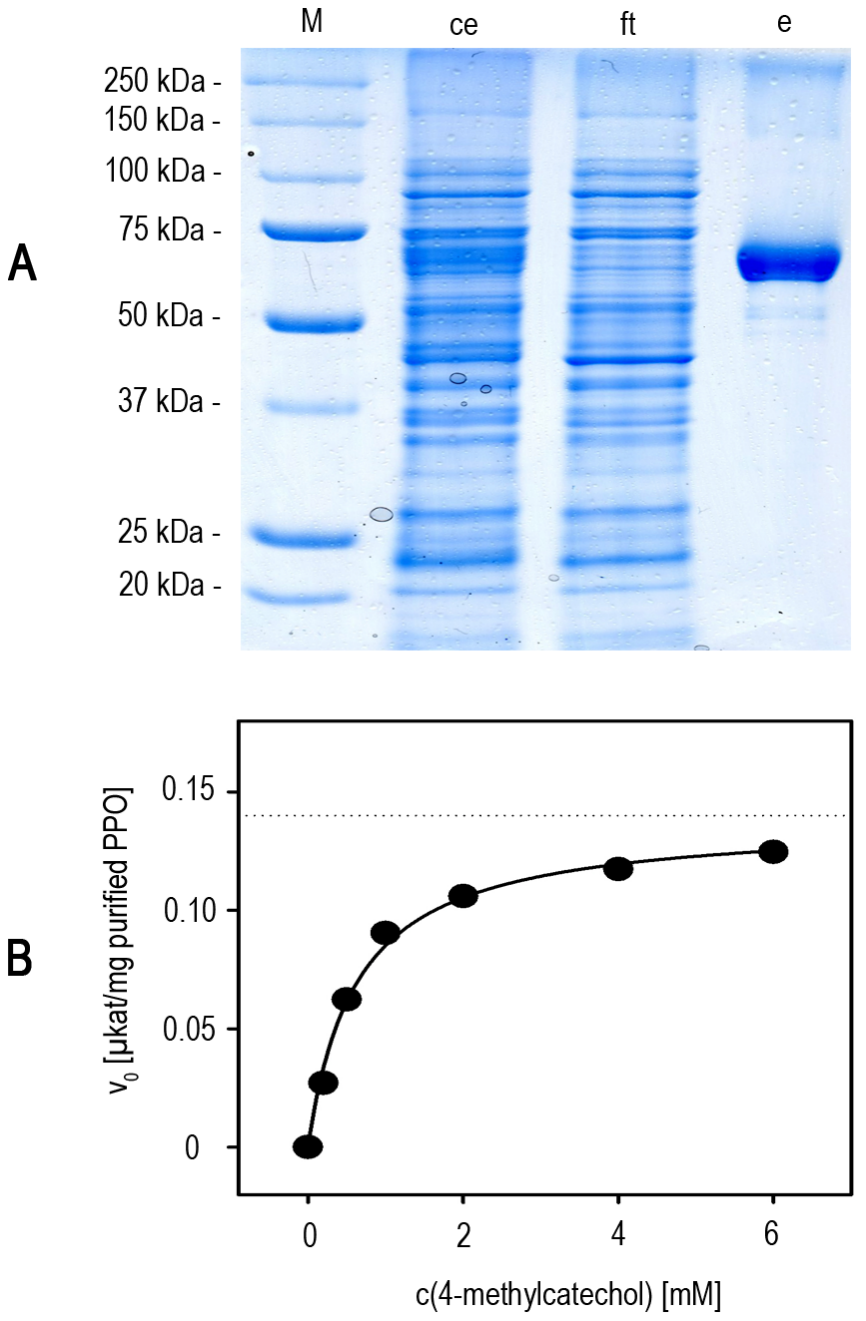
Heterologous expression of ToPPO-2. A Purification via Strep-Tactin-affinity chromatography. SDS-PAGE analysis was performed and proteins were stained by Coomassie Brilliant Blue. M: protein standard; ce: crude extract; ft: flow-through fraction; e: elution fraction. **B PPO-kinetics on 4-methylcatechol.** An example of a typical curve is shown. Non linear regression to the Michaels-Menten equation was performed (R^2^ = 0.99) to gain values for v_max_ (0.14±0.004 µkat per purified PPO; *dotted line*) and K_m_ (0.62±0.07 mM).

Using our established expression system (26°C, 48 h) we achieved average yields of 250±99 µg pure, fulllength ToPPO-2 protein – containing all three conserved PPO domains – from production cultures comprising 30 ml AIM supplemented with 0.02 mM CuCl_2_. Affinity chromatography using Strep-Tactin columns yielded sufficient amounts of highly pure and active ToPPO-2 for kinetic studies (Figure 3).

**Figure 4:**
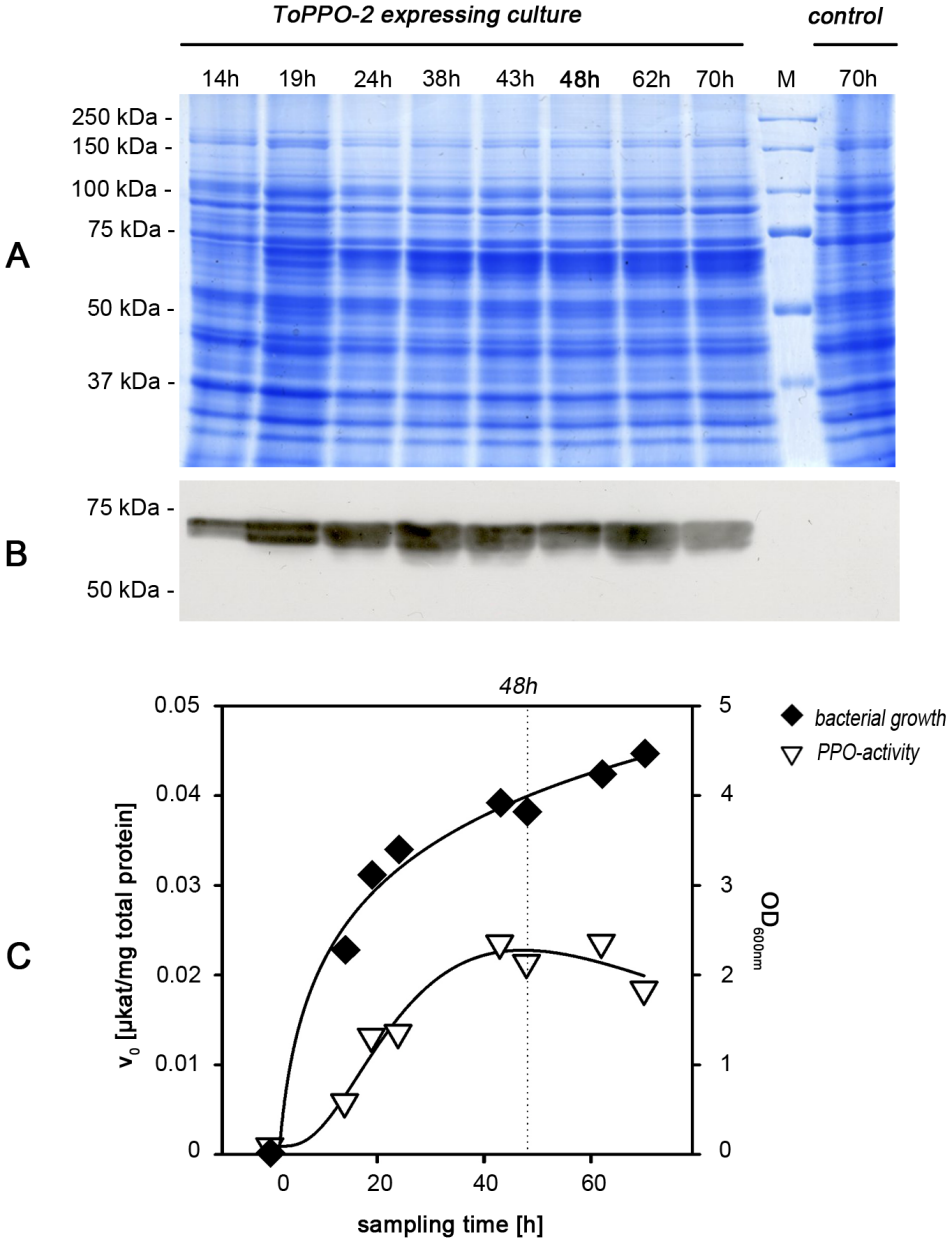
Optimal time for harvest of recombinant ToPPO-2. A SDS-PAGE of crude protein extracts. Samples were taken at different sampling times and analyzed by SDS-PAGE (same total protein amount per well). To visualize protein composition a Coomassie-staining was performed. **B Western blot analysis.** Proteins were blotted on a nitrocellulose membrane. Strep-Tactin-HRP conjugate was used to specifically detect the StrepII-tag of the recombinant PPO. **C Bacterial growth and PPO-activity over sampling time.** Samples were checked for their OD at 600 nm. Additionally crude protein extracts were analyzed for PPO-activity per mg of total protein. *The presented data show one experiment representative for three replicates*.

To verify the optimal harvesting time, we determined the OD_600 nm_ and PPO activity per mg protein in crude extracts of the production cultures, evaluating several time points over 70 h. As can be seen from Figure 4, the optimal harvesting time was at 48 h. PPO activity tended to decline after this point despite the increase in bacterial growth.

### Low Temperatures Increase the Yield of Active ToPPO-2

Recombinant proteins expressed in *E. coli* often fold more efficiently at low temperatures [20]. Our standard procedure for PPO expression involved continuous growth at 26°C, therefore we tested continuous growth at 20°C and 37°C, plus growth at 37°C until the culture reached an OD_600 nm_ value of 0.3 or 0.9 before transfer to 26°C. The latter approach was intended to accelerate growth and therefore biomass production before switching to the lower temperature after induction to promote protein folding at 26°C. Induction in AIM occurs automatically when the OD_600 nm_ reaches ∼1 [19].

**Figure 5:**
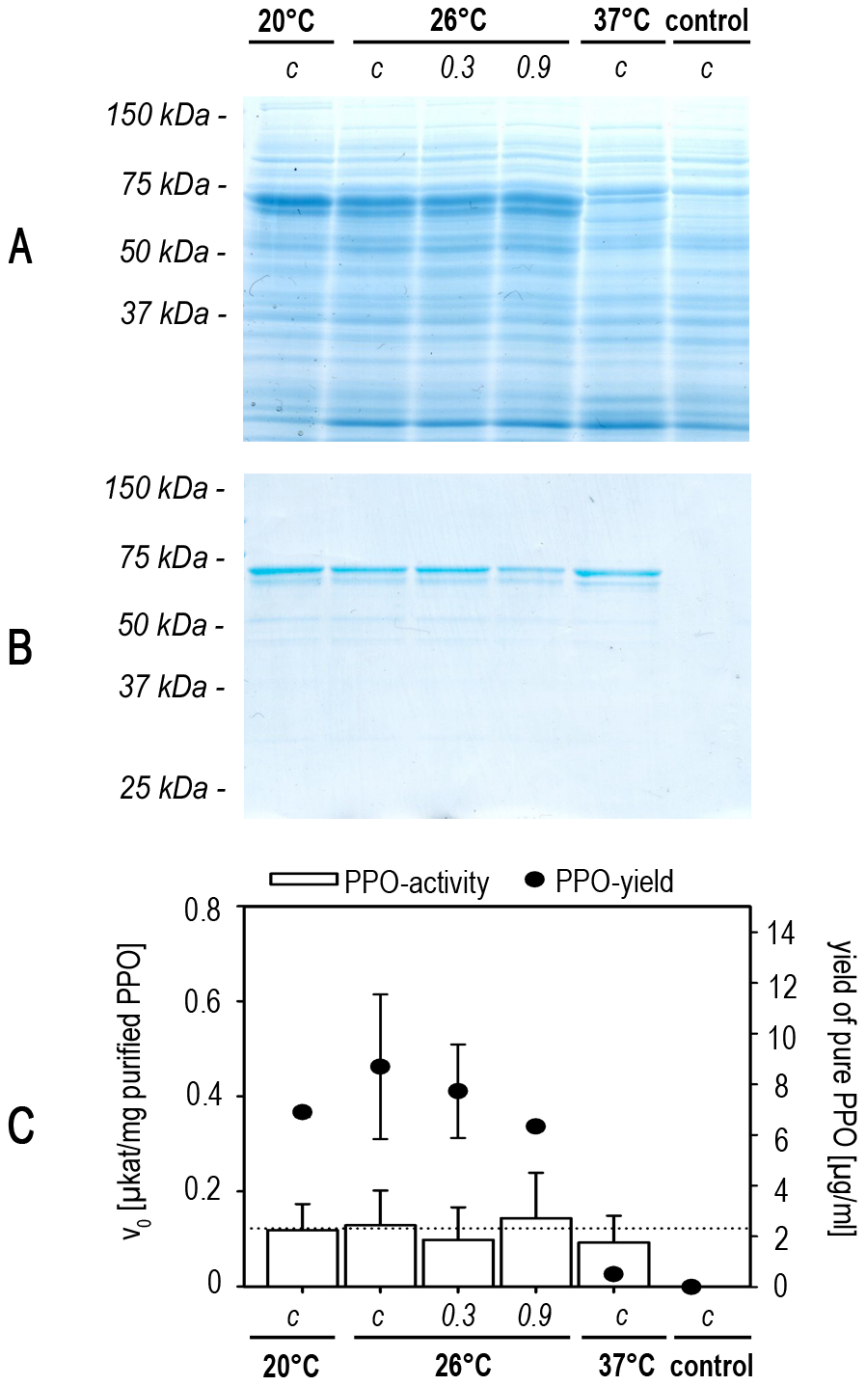
Optimal temperature for ToPPO-2 production. Production cultures were grown under different temperature settings. **A SDS-PAGE of crude protein extracts.** Samples were taken after 48 h and analyzed by SDS-PAGE (equal total protein amount per well). To visualize protein composition a Coomassie-staining was performed. **B SDS-PAGE of purified proteins.** ToPPO-2 was purified via affinity-chromatography using Strep-Tactin. Equal protein amounts were loaded on an acrylamide gel and analyzed by SDS-PAGE and Coomassie staining. **C Protein yields and specific PPO-activity after purification.** The specific PPO-activity (v_0_) was analyzed per mg of purified PPO. The average value from all samples is given as a dotted line. Yields of purified protein per volume of bacterial culture were also calculated. *c: continuous growth in given temperature; 0.3/0.9: growth at 37°C until OD_600 nm_ of 0.3/0.9 was reached, then grown at 26°C; control: empty vector control grown at 26°C.*

We evaluated the effect of these different cultivation temperatures on the expression of ToPPO-2 by monitoring the yields in crude protein extracts by SDS-PAGE (Figure 5 A). ToPPO-2 was purified (Figure 5 B) and we determined the specific activity per mg purified protein and yields of pure ToPPO per ml culture broth (Figure 5 C).

High yields of recombinant protein (8.7±2.9 µg per ml culture broth) and high specific activities (0.13±0.07 µkat per mg PPO-2) were achieved in the cultures grown continuously at 26°C. Higher temperature before induction or continuous growth at 20°C had no significant impact on the yield, whereas continuous growth at 37°C resulted in lower yields (6.2±1.7% of the yield from our standard conditions). In contrast to these impacts on PPO-yield, temperature had no significant effect on the specific activity of purified PPO, which remained at an average value of 0.12±0.07 µkat per mg protein.

Based on the results presented above, we therefore recommend continuous growth at 26°C for the optimal production of recombinant PPO, representing the best trade-off between protein yield and operational convenience. Continuous growth at 37°C substantially reduced the yield of soluble recombinant protein, confirming that folding is an important factor during the expression of recombinant PPOs in *E. coli*. The abundance of chaperones increases at low growth temperatures [20], suggesting that certain chaperones could be essential or at least supportive for correct PPO folding in *E. coli*. Furthermore, the positive impact of low growth temperatures may reflect the advantage of extending the time for protein folding given the lower growth rate of the bacteria. A combination of these effects (abundant chaperones and low growth rates) may promote efficient PPO folding in *E. coli*, as previously suggested for other proteins [27], [28].

### Adding Copper to the Growth Medium Promotes the Activity of ToPPO-2

Previous studies have recommended the addition of 0.02 mM CuCl_2_ to the growth medium during the bacterial expression of *Trifolium* PPO [6]. Because PPO requires two copper atoms to achieve correct folding of the active site and thus enzymatic activity, the addition of copper was adapted for our standard expression protocol [17]. Although the uptake of free copper is highly regulated in *E. coli* [29], [30], the supplementation of growth medium with copper may still increase the abundance of copper in the cytoplasm [27] and thus promote the correct folding of recombinant PPOs. We therefore tested the effect of different concentrations of CuCl_2_ on the yield of active ToPPO-2 in *E. coli* production cultures.

**Figure 6:**
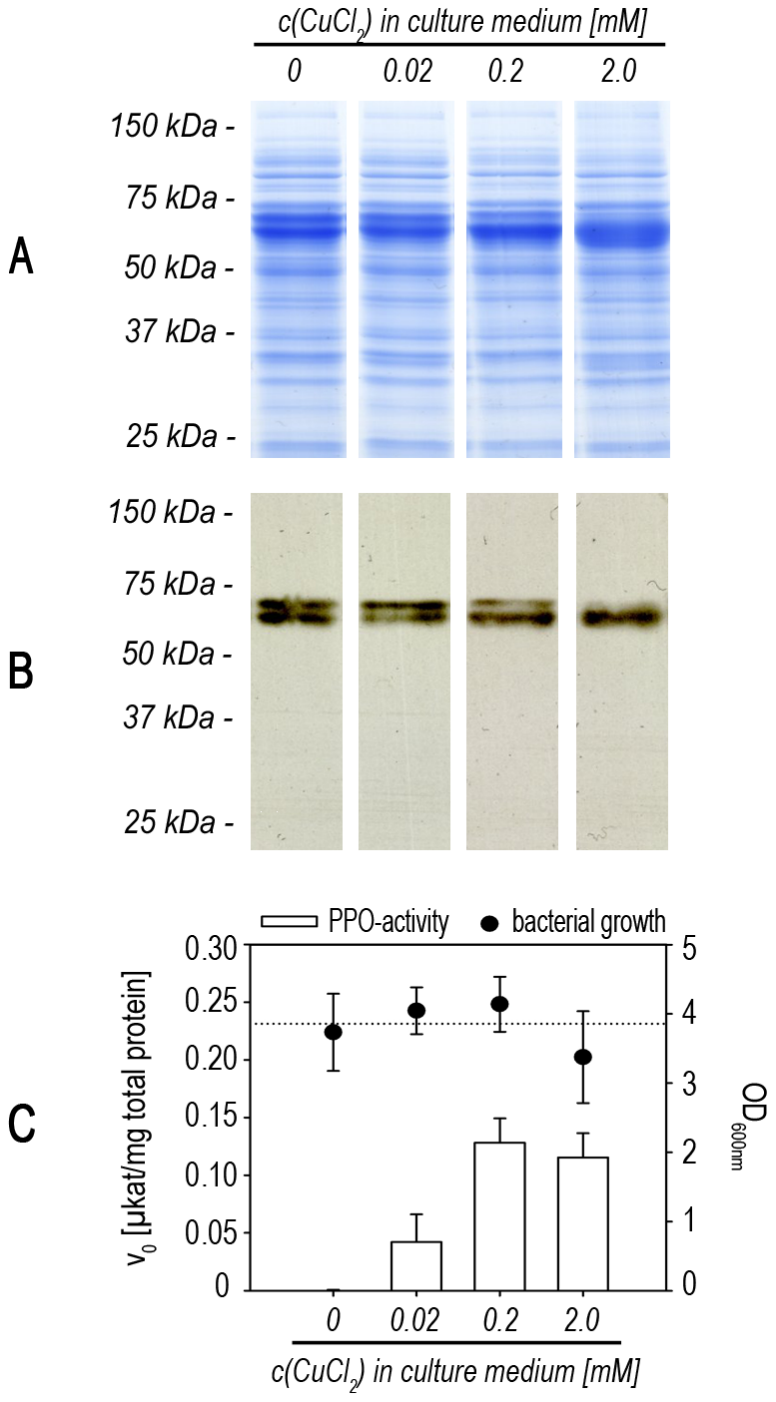
Dependency of PPO-expression on the amount of copper. Production cultures were grown with different amounts of CuCl_2_ supplemented in the AIM. **A SDS-PAGE of crude protein extracts.** Samples were taken after 48 h and analyzed by SDS-PAGE (equal total protein amount per well). To visualize protein composition a Coomassie-staining was performed. **B Western blot analysis.** Proteins were blotted on a nitrocellulose membrane. StrepTactin-HRP-conjugate was used to specifically detect the StrepII-tag of the recombinant PPO. **C Bacterial growth and PPO-activity.** Samples were checked for their OD at 600 nm. The average value from all samples is given as a dotted line. Additionally, crude protein extracts were analyzed for PPO-activity per mg of total protein.

Bacterial growth was affected only slightly (Figure 6 C) and the visible yield of recombinant protein did not change (Figure 6 A). However, the appearance of a double band following SDS-PAGE depended on the amount of copper in the growth medium (Figure 6 B) as did the PPO activity per mg total protein (Figure 6 C). The double band may indicate the presence of two folding isoforms as previously observed (Figure 4, Figure 5). Possibly, sections of the proteins – e.g. the copper-containing active site – remained partially folded and therefore influence the separation in SDS-PAGE.

**Figure 7:**
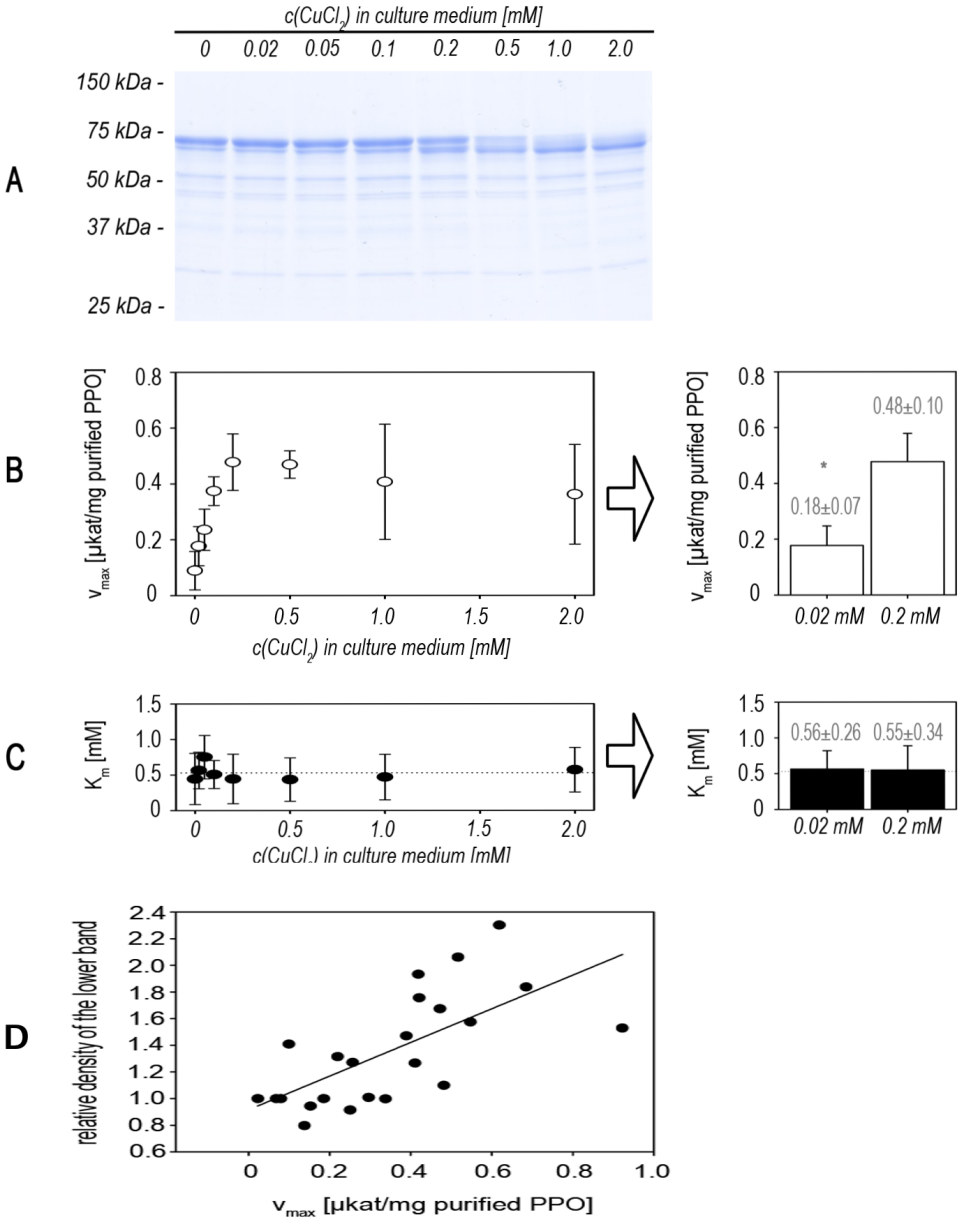
Influence of copper supplementation on enzymatic parameters. Production cultures were grown with different concentrations of supplemented CuCl_2_ in AIM. **A SDS-PAGE of purified proteins.** ToPPO-2 was purified via affinity-chromatography using Strep-Tactin. Equal protein amounts were loaded on an acrylamide gel and analyzed by SDS-PAGE and Coomassie-staining. **B Maximal activities (v_max_)** and **C Michaelis constants (K_m_).** Enzyme kinetics were performed on 4-methylcatechol as substrate and enzymatic parameters were determined by non-linear regression to the Michaelis-Menten equation. * P<0.01 in Student's t-test (n = 4); K_m_-values were not significantly different (P = 0.955). **D Correlation between the lower band appearing on SDS-PAGE and v_max_.** Data given are from three independent experiments. Analysis of the relative density of protein bands was performed using ImageJ [32] calculating the lower band in relation to the respective upper one. Poor separation of double bands at higher copper concentrations (1 – 2 mM) precluded their accurate quantification so that only data from the lower concentrations (0 – 0.5 mM) are included. *Statistics for correlation was performed using SigmaPlot11 and Pearson Product Moment Correlation, giving a significant positive correlation (corr. coefficient = 0.676, p<0.001, n = 23) between the tested parameters.*

After purification, the appearance of the lower band correlated strongly with increasing values for substrate turnover (v_max_) (Figure 7). The K_m_ values did not change significantly, strongly arguing that the ratios between active and inactive isoforms depend on the availability of copper. We assume that only the lower band represents the active, correctly folded copper-containing enzyme while the other band represents an incorrectly folded apo-form. This assumption is based on the obvious correlation between PPO-activity, copper loading and appearance of the lower band after SDS-PAGE.

**Figure 8:**
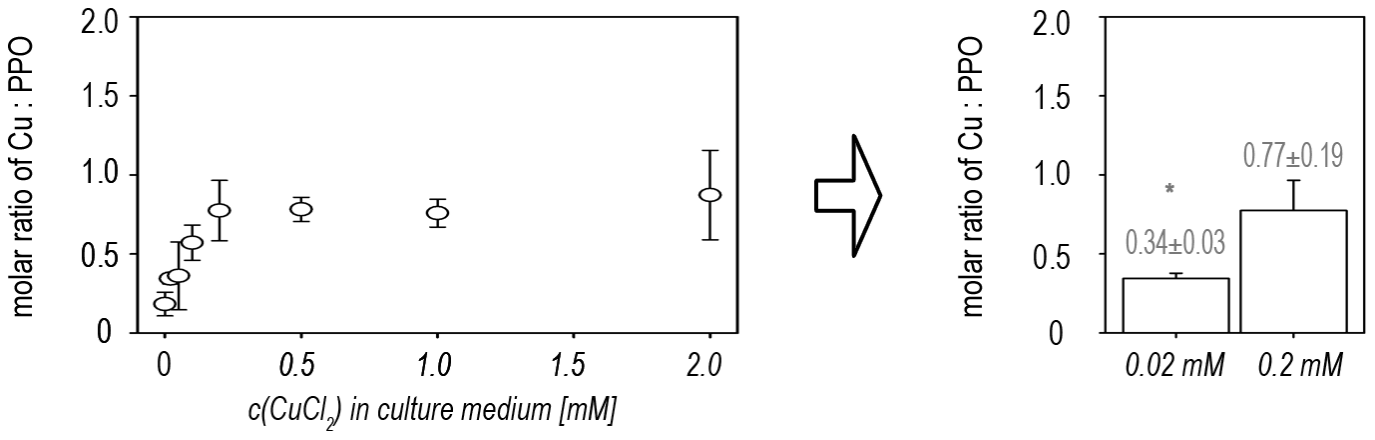
Copper-saturation of recombinant ToPPO-2. The copper content in aliquots containing defined amounts of purified ToPPO was determined using TXRF. Subsequently the molar ratio of Cu to purified PPO was calculated. * P<0.01 in Student's t-test (n = 4).

The amount of copper per mol of purified total PPO was determined by TXRF. Copper loading also increased in line with copper supplementation (Figure 8). The expected ratio of two copper atoms per mol of PPO was not achieved with the new method, indicating that copper loading remains suboptimal.

Our results confirm that the presence of 0.2 mM of CuCl_2_ in the bacterial growth medium is optimal for maximizing the yields of active ToPPO-2, resulting in a 2.7-fold increase in the yield of active protein compared to the original method, and achieving a specific turnover rate of 0.48±0.10 µkat per mg purified protein for the substrate 4-methylcatechol, as compared to 0.18±0.07 µkat per mg under the previously described standard conditions.

We hypothesize that the copper-resistance machinery in *E. coli* may support the proper folding of recombinant plant PPOs. It is unclear at present whether this reflects the greater abundance of copper in the cytoplasm due to the induction of copper transporters, or the more specific involvement of copper chaperones [29]. The combination of high temperatures and scarce copper resources in previous studies may have contributed to the lack of success in obtaining active recombinant PPOs.

### Our Novel Expression System is Transferable and Improves Activity of Further Plant PPOs like Tomato

**Figure 9:**
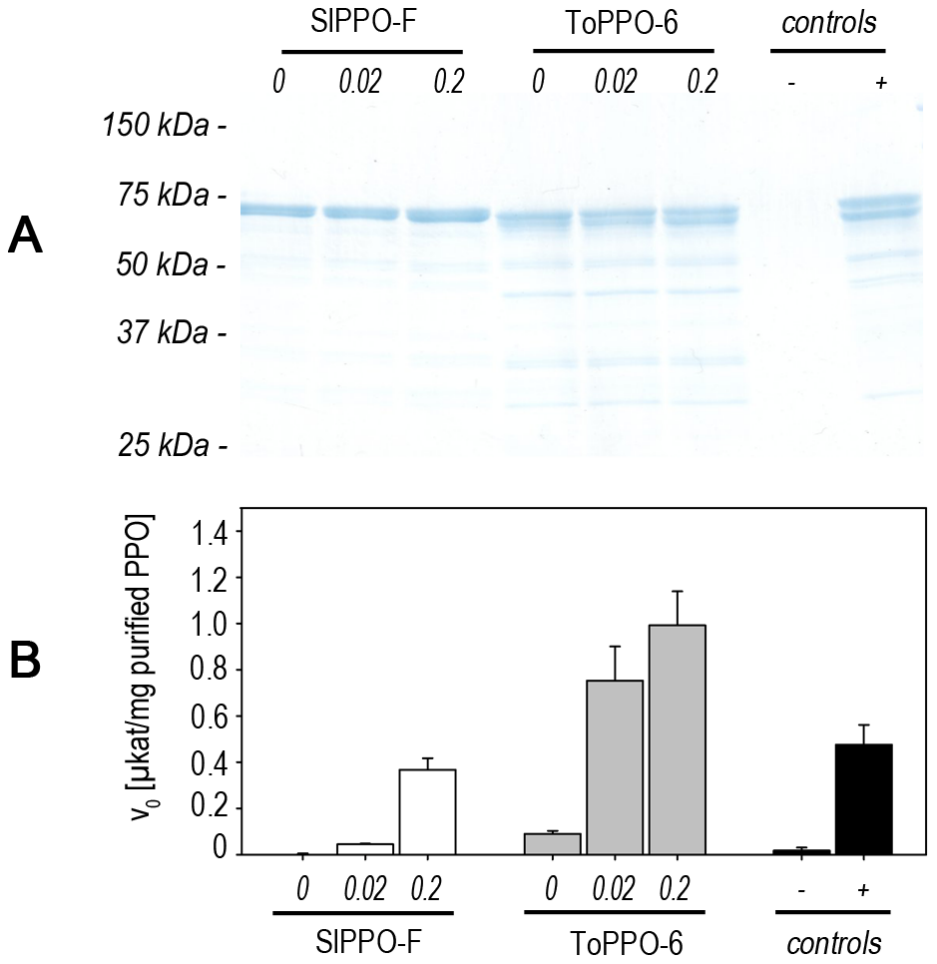
Transferability of the improved expression system to further eukaryotic PPOs. Further eukaryotic PPOs (s. Table 1) were cloned and heterologously expressed in *E. coli* Rosetta 2 (DE3) [pLysSRARE2]. Production cultures were grown with different concentrations (0 mM, 0.02 mM, 0.2 mM) of supplemented CuCl_2_ in AIM. **A SDS-PAGE of purified proteins.** Cultures were harvested after 48 h and recombinant PPOs were purified via affinity-chromatography using Strep-Tactin. Equal protein amounts were loaded on an acrylamide gel and analyzed via SDS-PAGE using Coomassie staining. **B Specific PPO-activity after purification.** Activity (v_0_) was analyzed per mg purified protein for 4-methylcatechol as substrate. *Purified ToPPO-2 produced with 0.2 mM CuCl_2_ supplementation served as positive control (+) and buffer as negative control (-).*

We cloned an additional PPO from a different plant source (Table 1) to test the transferability of the optimized expression system. This enzyme was also expressed successfully and purified in its active form (Figure 9). The impact of copper-supplemented growth media on the specific activity and the appearance of potential apo-forms of further PPOs was similar to our observations in experiments with dandelion PPO-2.

The successful expression of three different PPO genes with similarities of as low as 60% on nucleotide and 45% on protein level from two different species can be taken as evidence that our optimized bacterial expression system is suitable for the efficient synthesis of active PPOs from different plant species. It may be possible to increase the yields even further by optimizing the concentration of copper for different eukaryotic PPOs on a case by case basis.

## Conclusion

We have identified important parameters that enhance the bacterial production of active plant PPOs, i.e. a low cultivation temperature and the amount of copper provided during production. Our data indicate that folding and copper incorporation are major factors that limit the bacterial expression of plant PPOs. Even under our optimized conditions, copper loading remained suboptimal, and further research will therefore be necessary to elucidate the mechanisms and regulatory processes underlying heterologous and endogenous copper loading of PPOs. Our improved method allows the production of plant PPOs in sufficient amounts for enzyme characterization, and its simplicity and operational convenience will allow large-scale production e.g. for structural analysis by X-ray crystallography. Therefore this method shows great potential to facilitate PPO engineering, structural characterization, and functional analysis. Our data thus represent an important milestone on the way to a detailed understanding of type-3 copper proteins.

## Supporting Information

Figure S1 **Cleavage of the N-terminal StrepII-tag.** Aliquots of a sample of purified PPO were incubated at 4°C either with 0.1% (w/w) enterokinase (Ek) in 100 mM maleate-Tris buffer (pH 6.0) for 16 h or with buffer alone (control). **A SDS-PAGE after Enterokinase digestion.** To visualize protein composition Coomassie-staining was performed. **B Western blot analysis.** Proteins were blotted on a nitrocellulose membrane. Strep-Tactin-HRP conjugate was used to specifically detect the StrepII-tag of the recombinant PPO. **C PPO-activity after digestion.** The specific PPO-activity (v_0_) was analyzed per mg of purified PPO using 4 mM of 4-methylcatechol as a substrate.
(TIF)
