# Supplementary figures and images for: Parameters That Enhance the Bacterial Expression of Active Plant Polyphenol Oxidases
Source: PLoS One. 2013 Oct 21;8(10):e77291. doi: 10.1371/journal.pone.0077291 (PMC3804589; doi:10.1371/journal.pone.0077291)

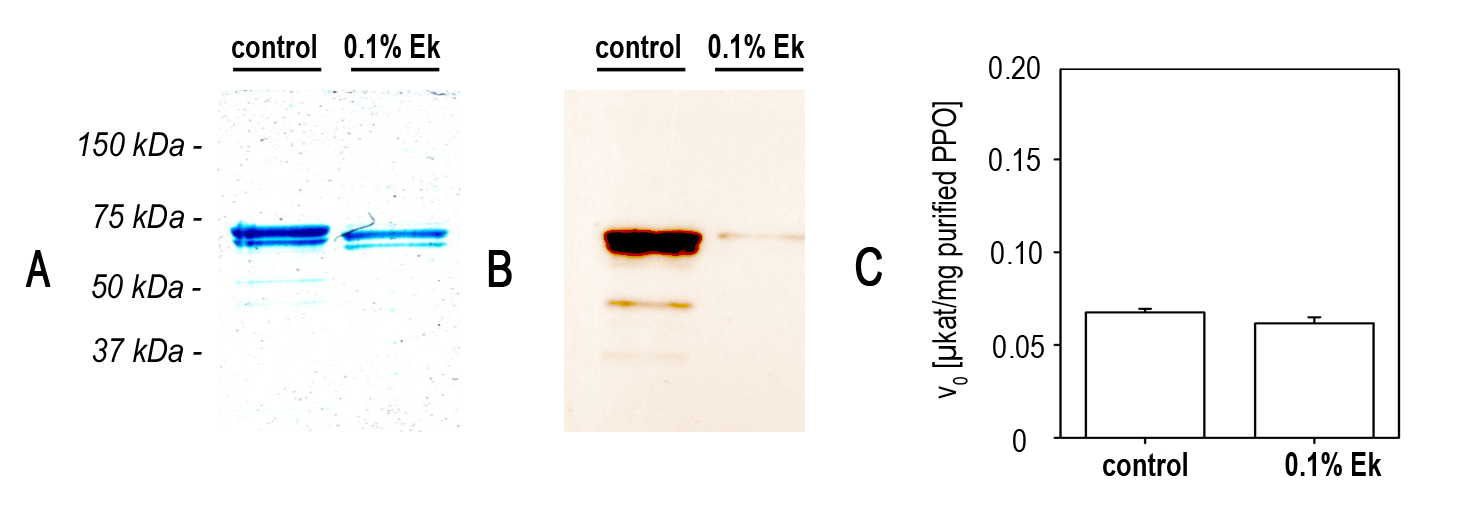

Supplement: Figure S1 — Cleavage of the N-terminal StrepII-tag. Aliquots of a sample of purified PPO were incubated at 4°C either with 0.1% (w/w) enterokinase (Ek) in 100 mM maleate-Tris buffer (pH 6.0) for 16 h or with buffer alone (control). A SDS-PAGE after Enterokinase digestion. To visualize protein composition Coomassie-staining was performed. B Western blot analysis. Proteins were blotted on a nitrocellulose membrane. Strep-Tactin-HRP conjugate was used to specifically detect the StrepII-tag of the recombinant PPO. C PPO-activity after digestion. The specific PPO-activity (v0) was analyzed per mg of purified PPO using 4 mM of 4-methylcatechol as a substrate. (TIF) [file pone.0077291.s001.tif]
